# Evaluation of Active Renin Concentration in A Cohort of Adolescents with Primary Hypertension

**DOI:** 10.3390/ijerph19105960

**Published:** 2022-05-13

**Authors:** Anna Deja, Piotr Skrzypczyk, Magdalena Nowak, Małgorzata Wrońska, Michał Szyszka, Anna Ofiara, Justyna Lesiak-Kosmatka, Anna Stelmaszczyk-Emmel, Małgorzata Pańczyk-Tomaszewska

**Affiliations:** 1 Department of Pediatrics and Nephrology, Doctoral School, Medical University of Warsaw, 02-091 Warsaw, Poland; adeja@wum.edu.pl (A.D.); michalszyszkaa@gmail.com (M.S.); 2 Department of Pediatrics and Nephrology, Medical University of Warsaw, 02-091 Warsaw, Poland; aniaofi@gmail.com (A.O.); mpanczyk1@wum.edu.pl (M.P.-T.); 3 Student Scientific Group, Department of Laboratory Diagnostics and Clinical Immunology of Developmental Age, Medical University of Warsaw, 02-091 Warsaw, Poland; magdalenawiktoria.nowak@gmail.com (M.N.); gosiawronska20@gmail.com (M.W.); 4 Student Scientific Group, Department of Pediatrics and Nephrology, Medical University of Warsaw, 02-091 Warsaw, Poland; justyna.lesiak@op.pl; 5 Department of Laboratory Diagnostics and Clinical Immunology of Developmental Age, Medical University of Warsaw, 02-091 Warsaw, Poland; anna.stelmaszczyk-emmel@wum.edu.pl

**Keywords:** renin, renin-angiotensin-aldosterone system, primary hypertension, isolated systolic hypertension, blood pressure, children

## Abstract

Our study aimed to assess active renin concentration in children with primary hypertension. Thus, we evaluated active renin concentration, clinical parameters, office and ambulatory blood pressure, and biochemical parameters in 51 untreated adolescents with primary hypertension (median: 14.4 [interquartile range—IQR: 13.8–16.8] years) and 45 healthy adolescents. Active renin concentration did not differ between patients with hypertension and healthy children (median: 28.5 [IQR: 21.9–45.2] vs. 24.9 [IQR: 16.8–34.3] [pg/mL], *p* = 0.055). In the whole group of 96 children, active renin concentration correlated positively with serum potassium and office and ambulatory systolic and diastolic blood pressures. Among children with hypertension, patients with isolated systolic hypertension had lower renin concentration than patients with systolic-diastolic hypertension (26.2 [IQR: 18.6–34.2] vs. 37.8 [IQR: 27.0–49.6] [pg/mL], *p* = 0.014). The active renin concentration did not differ between patients with isolated systolic hypertension and healthy children. In multivariate analysis, diastolic blood pressure Z-score (beta = 0.238, 95 confidence interval [0.018–0.458], *p* = 0.035) was the only predictor of active renin concentration in the studied children. We concluded that active renin concentration is positively associated with blood pressure and potassium in children, and diastolic blood pressure was the strongest predictor of renin level. Patients with isolated systolic hypertension may differ from patients with systolic-diastolic hypertension in less severe activation of the renin-angiotensin-aldosterone system.

## 1. Introduction

Arterial hypertension stands among the most common chronic diseases affecting society. Its prevalence is estimated at 3–5% in pediatric individuals [1]. Primary (essential) hypertension (PH) is the most common cause of elevated blood pressure in adult medicine and in adolescents after puberty [1,2].

The classic and probably the most studied pathophysiological pathway in hypertension, is the renin-angiotensin-aldosterone system (RAAS).

Renin is an aspartyl protease with a molecular weight of 37 kDa, produced in many tissues and organs. Still, the primary source of plasma renin is the juxtaglomerular apparatus and the collecting tubule cells. Renin is produced as prorenin, an inactive precursor with 386 amino acids [3]. In response to stimulating factors, active renin can be released either from a depot in the kidney or generated in the kidney by cleaving 43 amino acids at the N-terminus of prorenin by proprotein convertase 1. Prorenin secretion into the blood is continuous, in contrast to the tightly controlled release of renin, and blood concentration of prorenin is approximately tenfold higher than active renin [4].

The only known substrate of renin is angiotensinogen, from which the enzyme cleaves the 10-amino acid peptide angiotensin 1. In the classical RAAS axis, angiotensin one is converted by the angiotensin-converting enzyme (ACE) by removing two amino acids to angiotensin 2. Angiotensin II, a central player in the classical RAAS system, exerts biological actions by binding to its receptors [5]. Physiological responses to angiotensin receptor one stimulation by angiotensin II include tubular sodium reabsorption in the kidneys, aldosterone release in the adrenal glands, smooth muscle contraction in the arteries, stimulation of thirst and vasopressin production in the hypothalamus, in the medulla oblongata—activation of the sympathetic nervous system and decreased activity of the parasympathetic nervous system [3]. Besides the enzymatic action of soluble renin, binding of renin and prorenin to the membrane-bound prorenin/renin receptor in the brain, heart, placenta, liver, kidney, and pancreas enhances the efficiency of angiotensinogen cleavage. It induces angiotensin-independent intracellular prohypertensive and profibrotic effects by activating mitogen-activated kinases [6].

In clinical practice, the concentration of active renin or, requiring more restrictive sampling conditions, plasma renin activity, is most often determined. Single papers indicate that the measurement of active renin concentration is a specific marker of renin-angiotensin-aldosterone system activation, is more accurate at both low and high renin concentrations, and does not depend on angiotensinogen concentration [7,8].

Given the insufficient data in the pediatric population, our study aimed to evaluate the concentration of active renin in a population of untreated adolescents with primary hypertension and assess the relationship between active renin concentration, blood pressure, and selected clinical and biochemical parameters.

## 2. Materials and Methods

### 2.1. Study Group 

The study was a cross-sectional, single-center undertaking. Children and adolescents hospitalized between 2016 and 2019 at a single tertiary referral pediatric nephrology center specializing in diagnosing and treating arterial hypertension were eligible for the study. Inclusion criteria were untreated pharmacologically arterial hypertension diagnosed according to the European protocol [9], and exclusion criteria were the treatment with antihypertensive medications, lack of consent to participate in the study, chronic kidney disease, congenital or acquired heart defects or heart failure, and secondary forms of hypertension [9]. Finally, 51 children with untreated primary hypertension were enrolled in the study, and the control group consisted of 45 age- and sex-matched healthy children. The sample size was estimated based on the available literature on renin with a statistical power of 0.8, *p* = 0.05, and an effect size of 0.55 should be 45 [10,11,12,13,14,15].

### 2.2. Ethical Issues

The researchers obtained approval from the local bioethics committee to conduct the study (approval no. KB/58/2016, 15 March 2016). All procedures involving human participants were in accordance with the ethical standards of the institutional research committee and were performed in accordance with the declaration of Helsinki and its later amendments. All legal representatives of the patients and patients aged 16 years or older signed informed consent to participate in the study.

### 2.3. Clinical Parameters

All examinations were performed simultaneously on the same day using a unified protocol. The following clinical parameters were assessed: age, sex, and duration of hypertension. Basic anthropometric parameters were evaluated: height [cm], body weight [kg] and body mass index (BMI), and were presented as Z-scores (SDS) [16].

### 2.4. Active Renin Concentration

In all study participants, blood was collected after 2 h in an upright position in the morning (between 7 am and 10 am) after a 12-h fasting period according to our local protocol. All patients were evaluated in a euvolemic state on at least seven days of a normal sodium diet. Blood was allowed to clot, then promptly centrifuged under controlled conditions and frozen at −80 °C. Blood was thawed immediately before further analysis steps. The concentration of active renin [pg/mL] was determined by the ELISA (enzyme-linked immunosorbent assay) method (Labor Diagnostika Nord [LDN], Nordhorn, Germany) kit using a Biochrom Asys UVM 340 Scanning Microplate Reader (Biochrom Ltd., Cambridge, UK).

### 2.5. Other Laboratory Tests

Other parameters evaluated from peripheral blood included those assessed by standard local laboratory methods: morphology-based inflammatory parameters: neutrophil, lymphocyte, and platelet counts [1000/μL], mean platelet volume [fL], and calculated derivative indices: neutrophil-lymphocyte and platelet-lymphocyte ratio (NLR and PLR, respectively), serum creatinine concentration [µmol/L] used to calculate estimated glomerular filtration rate (eGFR) [17] [mL/min/1.73 m^2^], uric acid concentration [µmol/L], lipidogram: total, high-density lipoprotein (HDL), and low-density lipoprotein (LDL) cholesterol [mmol/L], and triglycerides [mmol/L], and serum sodium [mmol/L] and potassium [mmol/L]. In addition, we evaluated morning urinary albumin [g/L], sodium [mmol/L], potassium [mmol/L], and creatinine [mmol/L] with calculation of albumin-creatinine ratio [mg/g].

### 2.6. Blood Pressure

Blood pressure (BP) was assessed according to the pediatric protocol [9]. The analysis included oscillometrically measured office systolic and diastolic blood pressures (Welch Allyn VSM Patient Monitor 300 [Welch Allyn Inc., Skaneateles Falls, NY, USA]) as well as systolic, diastolic, and mean pressures (SBP, DBP, MAP) in ambulatory blood pressure monitoring (ABPM) (SUNTECH OSCAR 2 [SunTech Medical, Inc., Morrisville, NC, USA]). The 24-h blood pressure recordings allowed us to also analyze pressure loads during 24 h [%] and nocturnal dipping [%] together with pulse pressure (PP) [mm Hg] and heart rate (HR) [beats per minute—bpm]. The obtained values were interpreted using local norms for office BP [18] and global standards for ambulatory blood pressure monitoring (ABPM) [19]. Z-score (SDS) values were calculated for office and ambulatory 24-h blood pressure values.

### 2.7. Statistical Analysis

The obtained data were anonymized and archived (Excel 365, Microsoft 365, Microsoft, Redmond, WA, USA) and then statistically analyzed using Dell Statistica 13.0 PL software (TIBCO Software Inc., Palo Alto, CA, USA). First, the normality of data distribution was assessed using the Shapiro-Wilk test. Data depending on the distribution were presented as mean and standard deviation (SD) or median and interquartile range (IQR). We applied the following tests in the statistical analysis: Student’s t-test for independent groups, Pearson correlation, Mann-Whitney U test, and Spearman’s rank correlation. As active renin concentration had non-normal distribution, and non-parametric tests were used to assess its relation with other variables. Receiver operating characteristic (ROC) analysis was performed. The area under the curves (AUC) was calculated to determine the best cut-off values of renin. A multivariate analysis was performed using a general regression model. Parameters that correlated significantly with active renin concentration in univariate analysis were introduced into the model, excluding variables that correlated with each other with r > 0.600 to avoid collinearity. The results of multivariate analysis were presented as beta, confidence interval (CI), and a *p* value. A *p* value < 0.05 was used as the test of statistical significance.

## 3. Results

Clinical characteristics of the examined population are displayed in Table 1. There was no significant difference between children with arterial hypertension and the control group regarding sex and age. Both BMI and BMI Z-score were higher in children diagnosed with hypertension than in healthy children. The mean duration of hypertension was 16 months (median six months).

Biochemical characteristics of the population are featured in Table 2. There was no significant difference in active renin concentration between patients with hypertension and healthy children. On analysis of subclinical inflammation markers, hypertensive children were characterized by higher neutrophil count and neutrophil-lymphocyte ratio. Also, uric acid was higher in hypertensive patients. The only diversifying parameter was triglycerides, which were higher in adolescents with hypertension in terms of the lipid profile. Besides that, eGFR, total cholesterol, HDL, and LDL did not differ between the study and control groups. Serum sodium and potassium were significantly higher in hypertensive children, and so was the urinary sodium-creatinine ratio. Also, the urinary albumin-creatinine ratio was higher in the hypertensive group (Table 2).

Parameters concerning blood pressure—office measurements and ABPM results—are shown in Table 3. Both office and ambulatory pulse pressures were significantly higher in children with hypertension. Surprisingly, dipping status did not differ significantly between the two groups.

Active renin concentration did not differ between boys and girls in both the hypertensive group (32.3 [21.3–41.8] vs. 26.8 [23.1–45.6], *p* = 1.000 and in healthy patients (26.5 [15.5–34.3] vs. 23.9 [16.8–34.7], *p* = 0.702).

**Figure 1:**
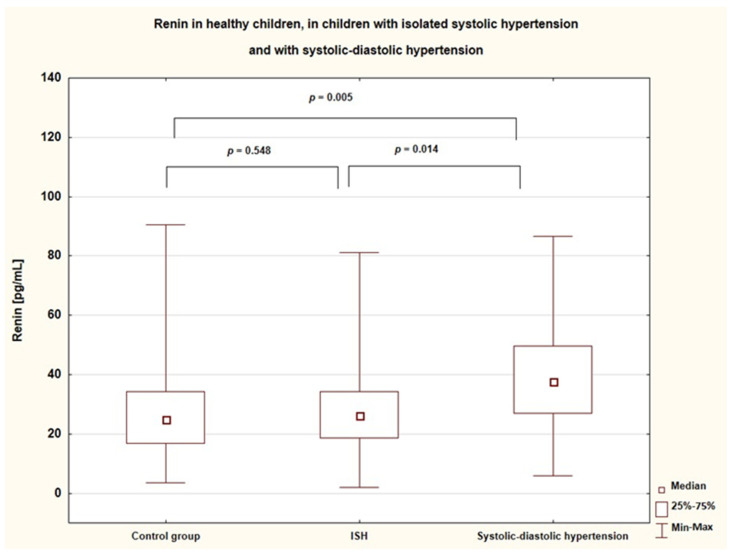
Active renin concentration in healthy children, in children with isolated systolic hypertension, and with systolic-diastolic hypertension (ISH—isolated systolic hypertension).

In the hypertensive group, patients suffering from both systolic and diastolic hypertension (*n* = 21) had significantly higher levels of active renin than patients diagnosed with isolated systolic hypertension (*n* = 30) (37.8 [27.0–47.6] vs. 26.2 [18.6–34.2], *p* = 0.014) and healthy children (*p* = 0.005). Patients with isolated systolic hypertension did not differ significantly in renin concentration from healthy subjects (*p* = 0.548). (Figure 1).

Table 4 includes correlations of various clinical, biochemical, and blood pressure parameters with serum active renin concentration. Considering the whole examined population, active renin concentration correlated positively with serum potassium level. No other significant correlations of renin and analyzed clinical and biochemical data were revealed. In the whole group and in hypertensive patients, we found numerous positive correlations between renin and office and ambulatory blood pressures. No such associations were demonstrated for healthy children.

**Figure 2:**
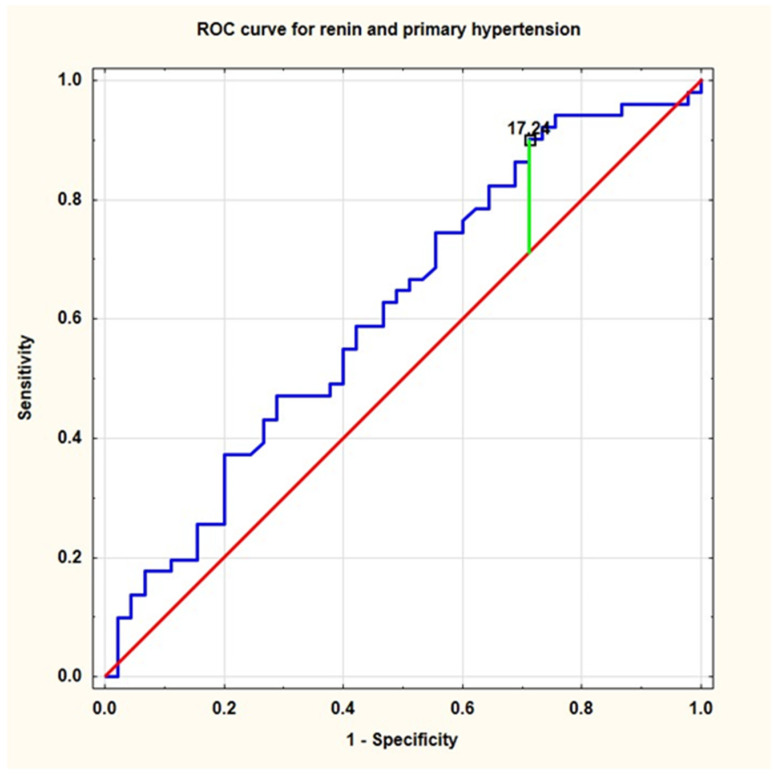
Receiver operating characteristic (ROC) curve for active renin concentration and the presence of primary hypertension (ROC—receiver operating characteristics).

**Figure 3:**
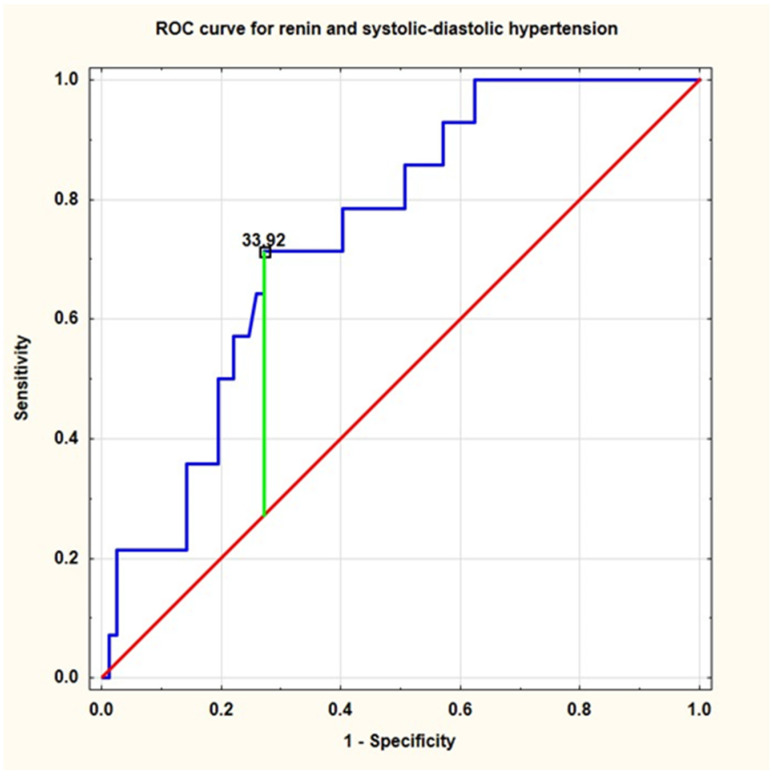
Receiver operating characteristic (ROC) curve for active renin concentration and the presence of systolic-diastolic hypertension (ROC—receiver operating characteristics).

The results of ROC analysis was presented in Table 5. ROC analysis demonstrated good diagnostic profiles (AUC, sensitivity and specificity) for the presence of primary hypertension, systolic-diastolic hypertension, and elevated (≥95th percentile) (both office and ambulatory) blood pressure. The cut-off value of active renin concentration to predict hypertension was 17.2, and to predict systolic-diastolic hypertension it was 33.9 [pg/mL], respectively (Figure 2 and Figure 3, respectively).

In multivariate analysis, diastolic blood pressure Z-score (beta = 0.238, 95CI [0.018–0.458], *p* = 0.035) was the only predictor of active renin concentration in the studied children.

## 4. Discussion

Our cross-sectional study evaluated active renin concentrations in a large population of pediatric patients with primary hypertension and compared it with healthy peers. We demonstrated no significant differences in renin levels between children with hypertension and healthy children. When compared to healthy peers, renin was significantly higher in children with systolic-diastolic hypertension, but was comparable in children with isolated systolic hypertension. In the cohort of 99 children analyzed, renin correlated positively with blood pressure and serum potassium level. Diastolic blood pressure was the only significant predictor of renin level in a multivariate analysis.

Measuring active instead of total renin and prorenin concentration or plasma renin activity has been performed for almost 30 years. On the one hand, the determination of plasma renin activity requires strict conditions (transport in ice to prevent activation and consumption of the enzyme). On the other hand, it is dependent on the amount of substrate in plasma (angiotensinogen). The assay may be inadequate in situations with high and low renin concentrations. Previously, the active renin concentration was determined using immunoradiometric assays. The present study used a simple ELISA technique. This is a promising technique replacing, in many laboratories, the technically demanding plasma renin activity assessment. Of note, international recommendations allow direct measurement of active renin concentration and plasma renin activity to diagnose primary aldosteronism and monogenic forms of hypertension [20,21].

First of all, our study revealed significant though weak positive correlations between active renin concentration and office systolic and diastolic blood pressure expressed both as [mm Hg] and Z-scores both in the whole participants’ cohort and among patients with hypertension, but no such association. The relationship between blood pressure and renin in healthy individuals and hypertensive patients may differ. It may reflect the complex role of RAAS in the pathophysiology of primary hypertension. For example, no significant relation between components of RAAS and blood pressure was found in a Chinese cohort of pediatric patients with primary hypertension [13]. On the contrary, Shatat and Flynn demonstrated a negative relationship between plasma renin activity and 24-h systolic blood pressure and 24-h pulse pressure in obese adolescents [11]. In the authors’ opinion, the latter finding could be a consequence of the putative insulin-mediated renin-independent sodium reabsorption, volume expansion, and renin inhibition in obesity. In young, healthy 1353 adults aged 25–41 years, blood pressure was directly related to aldosterone, the aldosterone-renin ratio, and inversely to renin concentration [15]. The authors hypothesized that blood pressure rise could be driven in this cohort by a renin-independent aldosterone mechanism and some of the studied patients could have unrecognized primary aldosteronism. On the other hand, despite inconsistent results on the mutual relationship between blood pressure and renin, numerous data indicate that increased renin is an independent risk factor for cardiovascular events in adult populations [10,14].

Our results might suggest that renin-angiotensin-aldosterone system activation could be one of the driving forces of blood pressure elevation in the young. Of note, secondary forms of hypertension were excluded in all of our patients. Thus, there were no patients with typically renin-mediated hypertension (e.g., unilateral renal artery stenosis) or low-renin hypertension (e.g., monogenic hypertension or primary aldosteronism).

The significant difference in active renin concentration between untreated patients with isolated systolic hypertension and systolic-diastolic hypertension is a remarkable finding of this study. Isolated systolic hypertension has become a hot topic in pediatric and young adult hypertension in recent years. First, it is the predominant phenotype of hypertension in this age group. Second, increasing data indicate that these patients are significantly different from patients with systolic-diastolic hypertension. The isolated increase in systolic blood pressure in young individuals is not apparent. Postulated pathways include hyperkinetic circulation and high aortic elasticity leading to significant amplification of peripheral arterial pressure [22]. And indeed, despite the fact that isolated systolic hypertension seems to be a heterogeneous condition, many of these patients have normal central blood pressure (“spurious hypertension”) and show no hypertension-mediated organ damage [23]. Our results may also indicate that, unlike patients with systolic-diastolic hypertension, they may not present with renin-angiotensin-aldosterone system activation. It can be presumed that an angiotensin-mediated increase in peripheral resistance may not occur in this group of patients, or this activation is low-grade and therefore nondetectable in our study, which was not powered to answer this specific question. Multivariate analysis showed the strongest association between renin levels and diastolic blood pressure. Diastolic pressure is determined mainly by total peripheral resistance, which is significantly influenced by angiotensin II-induced vasoconstriction [24]. Otherwise, systolic blood pressure depends largely on renin-independent factors such as cardiac output and the elastic properties of large arteries [22]. This may explain the observed difference between the two groups of patients with essential hypertension. Indeed, further studies, including prospective analysis of the renin-angiotensin-aldosterone system in patients with isolated systolic hypertension, are needed. Only some of these patients progress to sustained hypertension [25]. It is also necessary to search for the relationship between the RAAS, central blood pressure, and peripheral resistance in young patients with hypertension.

Traditionally, activation of the renin-angiotensin-aldosterone system promotes renal tubular sodium reuptake and urinary potassium wasting. Both aldosterone and angiotensin II have their receptors on distal tubular cells influencing ion homeostasis [3]. Thus, a weak positive correlation between renin concentration and potassium may seem surprising. Hypertension forms typically associated with marked activation of the renin-angiotensin-aldosterone system, such as unilateral renal artery stenosis, are often associated with hypokalemia [9]. On the other hand, with preserved ion homeostasis, the physiological response to an increase in serum potassium concentration is to activate mechanisms aimed at removing the excess of this ion. In a classic Japanese study in patients with primary hypertension remaining on a high-sodium diet, the addition of potassium chloride resulted in a reduction in blood pressure but also in an increase in plasma renin activity [26]. Since that time, numerous studies have examined the relationship between sodium and dietary potassium intake, serum and urine levels, and components of RAAS in both normotensive and hypertensive individuals, bringing inconsistent results [27]. All our patients remained on a normal sodium diet without specific recommendations for potassium intake. Of note, we found no relationship between renin concentration and urinary sodium and potassium in our cohort. However, the latter parameter was evaluated in a small group of patients and controls. Finally, it should be noted that the correlation of renin with serum potassium was weak (r = 0.228), was not present in the subgroups, and does not appear to be clinically significant, as all patients had normal potassium levels. Furthermore, the correlation disappeared in the multivariate analysis.

Kidney-independent activation of RAAS in adipose tissue has been proposed to link obesity and the risk of hypertension. Expression of virtually all components of RAAS has been demonstrated in adipocytes, and adipose-derived angiotensinogen is released into plasma, thus contributing to an increase in systemic blood pressure [28]. Conversely, RAAS has essential effects on metabolism, including lipid metabolism. In vitro, angiotensin II stimulated lipogenesis in human adipocytes [29]. The same molecule can induce insulin resistance and inhibit adiponectin secretion [30]. The lack of correlations between lipid parameters and renin stands in opposition to numerous adult data [10,12,14]. We cannot exclude the possibility that in children and adolescents with hypertension, overactivity of the renin-angiotensin-aldosterone system leads to significant sympathetic activation, which could promote lipolysis and offset the negative effects of the RAA system in young people on lipid parameters. On the other hand, the methodological differences in renin evaluation should be considered as possible causes of different results between the studies (immunochemiluminescent method in PREVEND [14], plasma renin activity in HOPE [10] and in the Polish [12] adult studies). Finally, our study was not powered to investigate this specific question and this lack of correlation may simply reflect an unpowered analysis.

We found no association between active renin concentration and BMI in our cohort. In the previously cited Shatat and Flynn cohort, plasma renin activity correlated positively with BMI [11]. The difference in the results of our study and the Shahat and Flynn study may reflect the already discussed difference in methodology between renin concentration and plasma renin activity determination. Plasma renin activity depends on the concentration of angiotensinogen, one source in plasma is adipose tissue, which is not significant as a source of circulating renin.

It is essential to emphasize the limitations of this study. First, the study’s cross-sectional nature makes it impossible to infer causal relationships between renin and blood pressure levels. Second, we did not compare values of active renin concentration with the assay used in clinical practice—plasma renin activity or aldosterone levels—as most patients did not have this test done (the results were accessible only for eight hypertensive children)—in our center, they are not routinely performed in adolescents with stage I hypertension and the absence of end-organ damage. An important limitation of the study is the lack of data on urinary potassium excretion in most patients studied. In addition, we used local standards to analyze blood pressure, which may limit the relevance of our results to other pediatric populations. Finally, the study did not analyze end-organ damage or central blood pressure.

## 5. Conclusions

Despite significant advances in knowledge regarding the pathogenesis of primary hypertension, the renin-angiotensin-aldosterone system remains at the heart of various pathophysiological concepts. Our results among children and adolescents with primary hypertension indicate that the concentration of active renin is positively associated with blood pressure elevation and serum potassium level. Interestingly, this study also revealed no increase in renin in patients with isolated systolic hypertension, and diastolic blood pressure was the only predictor of renin concentration in multivariate analysis. This finding might help to understand the differences between isolated systolic hypertension and systolic-diastolic hypertension in the young.

## Figures and Tables

**Table 1:** Clinical characteristics of the examined population.

Parameter	Children with Primary Hypertension	Control Group	*p*
Number of patients (*n)*	51	45	-
Sex (boys/girls) (*n*)	34/17	27/18	*p* = 0.498
Age [years]	14.4 (13.8–16.8)	14.6 (12.4–16.6)	*p* = 0.436
BMI [kg/m^2^]	24.9 ± 5.1	21.9 ± 4.6	*p* = 0.006
BMI Z-score	1.2 (0.4–1.7)	0.5 (−0.6–1.3)	*p* = 0.003
Duration of hypertension [mths]	6.0 (3.0–18.0)	-	-

BMI—body mass index.

**Table 2:** Biochemical characteristics of the examined population.

Parameter	Children with Primary Hypertension	Control Group	*p*
Active renin concentration [pg/mL]	28.5 (21.9–45.2)	24.9 (16.8–34.3)	*p* = 0.055
Neutrophils [1000/µL]	3.5 (2.5–4.3)	2.6 (2.2–3.2)	*p* < 0.005
Lymphocytes [1000/µL]	2.2 (1.7–2.6)	2.2 (1.9–2.6)	*p* = 0.666
Platelets [1000/µL]	269.1 ± 60.5	253.3 ± 55.1	*p* = 0.238
NLR	1.5 (1.1–1.9)	1.2 (0.9–1.5)	*p* = 0.002
PLR	122.0 (99.2–150.0)	121.2 (89.4–139.8)	*p* = 0.295
MPV (fL)	10.0 (8.9–11.1)	10.6 (10.2–11.1)	*p* = 0.082
eGFR [mL/min/1.73 m^2^]	93.4 (81.2–116.0)	101.5 (91.1–113.6)	*p* = 0.215
Uric acid [µmol/L]	327.1 ± 89.2	303.3 ± 89.2	*p* = 0.046
Total cholesterol [mmol/L]	4.0 (3.5–4.6)	4.1 (3.5–4.6)	*p* = 0.907
LDL cholesterol [mmol/L]	2.2 (1.7–2.7)	2.1 (1.7–2.8)	*p* = 0.728
HDL cholesterol [mmol/L]	1.3 (1.1–1.6)	1.4 (1.1–1.8)	*p* = 0.119
Triglicerides [mmol/L]	1.0 (0.7–1.5)	0.7 (0.6–1.0)	*p* = 0.004
Serum sodium [mmol/L]	143.0 (142.0–144.0)	142.0 (140.0–143.0)	*p* = 0.005
Serum potassium [mmol/L]	4.6 (4.3–4.7)	4.4 (4.1–4.6)	*p* = 0.014
Urinary sodium/ creatinine ratio	13.1 (9.3–19.0)	9.9 (6.0–14.4)	*p* = 0.035
Urinary potassium/creatinine ratio	5.0 (3.2–7.9) *	2.9 (2.1–4.5) *	*p* = 0.080
Urinary sodium/ potassium ratio	2.3 (2.0–3.6) *	3.5 (2.4–5.1) *	*p* = 0.251
Urinary Alb/crea ratio [mg/g]	9.3 (5.1–12.3)	4.7 (3.2–8.4)	*p* = 0.008

NLR—neutrophil-to-lymphocyte ratio, PLR—platelet-to-lymphocyte ratio, MPV—mean platelet volume, eGFR—estimated glomerular filtration rate according to Schwartz formula, LDL—low-density lipoprotein, HDL—high-density lipoprotein, Alb/crea ratio—albumin-to-creatinine ratio in urine. *—evaluated in 11 children with primary hypertension and 16 healthy controls.

**Table 3:** Arterial hypertension in the examined population—office measurement and ambulatory blood pressure monitoring parameters.

Parameter	Children with Primary Hypertension	Control Group	*p*
**Office Blood Pressure Measurement**			
SBP [mm Hg]	144.4 ± 11.1	122.3 ± 14.6	*p* < 0.001
SBP Z-score	2.3 (1.8–3.3)	0.8 (0.1–1.6)	*p* < 0.001
DBP [mm Hg]	82.0 (78.0–92.0)	72.0 (65.0–78.0)	*p* < 0.001
DBP Z-score	1.5 (0.9–2.2)	0.4 (−0.1–0.9)	*p* < 0.001
Pulse pressure [mm Hg]	57.0 ± 10.6	50.5 ± 9.7	*p* = 0.002
**24-h Ambulatory Blood Pressure Measurement**			
SBP 24 h [mm Hg]	133.0 (130.0–136.0)	117.0 (114.0–121.0)	*p* < 0.001
SBP 24 h Z-score	2.1 (1.6–2.7)	0.3 (−0.4–0.7)	*p* < 0.001
DBP 24 h [mm Hg]	72.7 ± 6.9	65.6 ± 4.3	*p* < 0.001
DBP 24 h Z-score	0.9 ± 1.3	−0.4 ± 0.9	*p* < 0.001
MAP 24 h [mm Hg]	92.6 ± 6.2	82.6 ± 4.5	*p* < 0.001
MAP 24 h Z-score	1.4 (1.0–2.1)	0.0 (−0.6–0.6)	*p* < 0.001
PP 24 h [mm Hg]	60.2 ± 7.4	51.2 ± 5.6	*p* < 0.001
HR 24 h [bpm]	79.078.0 (71.0–89.0)	74.0 (67.0–83.0)	*p* = 0.238
HR 24 h Z-score	−0.1 ± 1.3	−0.5 ± 1.3	*p* = 0.127
SBPL/24 h (%)	49.0 (36.0–64.0)	20.0 (6.0–19.0)	*p* < 0.001
DBPL/24 h (%)	22.0 (17.0–38.0)	10.0 (4.0–12.0)	*p* < 0.001
Systolic DIP (%)	12.0 (8.2–13.8)	11.5 (7.1–14.1)	*p* = 0.871
Diastolic DIP (%)	15.7 (11.0–22.1)	16.1 (11.6–19.0)	*p* = 0.972

SBP—systolic blood pressure, DBP—diastolic blood pressure, MAP—mean arterial pressure, PP—pulse pressure, HR—heart rate, SBPL—systolic blood pressure load, DBPL—diastolic blood pressure load, DIP–blood pressure dipping.

**Table 4:** Correlations of serum active renin concentration (ARC) with clinical and biochemical parameters in the examined population (R, P).

Parameter Correlating with ARC	All Study Participants (*n* = 99)	Patients with Primary Hypertension (*n* = 51)	Control Group (*n* = 45)
**Clinical Parameters**			
Age [years]	R = −0.108 *p* = 0.293	R = −0.112 *p* = 0.434	R = −0.119 *p* = 0.435
BMI [kg/m^2^]	R = −0.140 *p* = 0.177	R = −0.149 *p* = 0.296	R = −0.233 *p* = 0.132
BMI Z-score	R = −0.047 *p* = 0.650	R = −0.053 *p* = 0.713	R = −0.195 *p* = 0.204
Duration of hypertension [months]	-	R = −0.163 *p* = 0.264	-
**Laboratory Parameters**			
Neutrophils [1000/µL]	R = 0.052 *p* = 0.618	R = −0.074 *p* = 0.607	R = 0.052 *p* = 0.735
Lymphocytes [1000/µL]	R = 0.001 *p* = 0.989	R = 0.037 *p* = 0.798	R = −0.058 *p* = 0.705
Platelets [1000/µL]	R = 0.101 *p* = 0.326	R = −0.181 *p* = 0.204	R = −0.064 *p* = 0.679
NLR	R = 0.039 *p* = 0.707	R = −0.088 *p* = 0.541	R = 0.072 *p* = 0.638
PLR	R = −0.072 *p* = 0.486	R = −0.134 *p* = 0.348	R = −0.022 *p* = 0.885
MPV (fL)	R = 0.046 *p* = 0.656	R = 0.230 *p* = 0.104	R = −0.095 *p* = 0.533
eGFR [mL/min/1.73 m^2^]	R = 0.065 *p* = 0.530	R = 0.003 *p* = 0.986	R = 0.183 *p* = 0.234
Uric acid [µmol/L]	R = −0.066 *p* = 0.534	R = −0.142 *p* = 0.336	R = −0.052 *p* = 0.736
Total cholesterol [mmol/L]	R = −0.099 *p* = 0.350	R = −0.128 *p* = 0.382	R = −0.107 *p* = 0.493
LDL cholesterol [mmol/L]	R = −0.135 *p* = 0.207	R = −0.136 *p* = 0.367	R = −0.132 *p* = 0.397
HDL cholesterol [mmol/L]	R = −0.097 *p* = 0.364	R = −0.158 *p* = 0.288	R = −0.024 *p* = 0.878
Triglycerides [mmol/L]	R = 0.027 *p* = 0.796	R = −0.044 *p* = 0.763	R = −0.026 *p* = 0.866
Serum sodium [mmol/L]	R = 0.074 *p* = 0.475	R = −0.085 *p* = 0.557	R = 0.168 *p* = 0.269
Serum potassium [mmol/L]	**R = 0.225 *p* = 0.028**	R = 0.267 *p* = 0.061	R = 0.127 *p* = 0.404
Urinary sodium/ crea ratio	R = 0.146, *p* = 0.224	R = −0.017 *p* = 0.919	R = 0.218 *p* = 0.230
Urinary potassium/crea ratio	R = 0.174, *p* = 0.384	R = −0.154 *p* = 0.650	R = 0.127 *p* = 0.641
Urinary sodium/ potassium	R = 0.017, *p* = 0.560	R = 0.209, *p* = 0.537	R = 0.174, *p* = 0.520
Alb/Crea ratio [mg/g]	R = 0.106 *p* = 0.374	R = 0.028 *p* = 0.808	R = −0.152 *p* = 0.422
**Office Blood Pressure**			
SBP [mm Hg]	**R = 0.248 *p* = 0.016**	R = 0.142 *p* = 0.320	R = 0.110 *p* = 0.482
SBP Z-score	**R = 0.347 *p* < 0.001**	**R = 0.289 *p* = 0.040**	R = 0.199 *p* = 0.200
DBP [mm Hg]	**R = 0.243 *p* = 0.018**	R = 0.194 *p* = 0.174	R = 0.092 *p* = 0.559
DBP Z-score	**R = 0.319 *p* = 0.002**	R = 0.301 *p* = 0.032	R = 0.152 *p* = 0.330
PP [mm Hg]	R = 0.077 *p* = 0.462	R = −0.045 *p* = 0.654	R = 0.152 *p* = 0.331
**24-h Ambulatory Blood Pressure**			
SBP 24 h	**R = 0.185 *p* = 0.080**	R = 0.147 *p* = 0.314	R = −0.004 *p* = 0.982
SBP 24 h Z-score	**R = 0.221 *p* = 0.035**	R = 0.191 *p* = 0.188	R = −0.004 *p* = 0.979
DBP 24 h	**R = 0.228 *p* = 0.030**	R = 0.271 *p* = 0.060	R = 0.021 *p* = 0.896
DBP 24 h Z-score	**R = 0.241 *p* = 0.022**	R = 0.280 *p* = 0.051	R = 0.066 *p* = 0.676
MAP 24 h	**R = 0.221 *p* = 0.036**	R = 0.270 *p* = 0.061	R = −0.025 *p* = 0.876
MAP 24 h Z-score	**R = 0.263 *p* = 0.012**	R = 0.261 *p* = 0.070	R = 0.047 *p* = 0.767
PP 24 h	**R = 0.010 *p* = 0.921**	R = −0.133 *p* = 0.364	R = −0.058 *p* = 0.714
HR 24 h	**R = 0.289 *p* = 0.005**	R = 0.239 *p* = 0.098	R = 0.287 *p* = 0.065
HR 24 h Z-score	**R = 0.292 *p* = 0.005**	R = 0.247 *p* = 0.087	R = 0.275 *p* = 0.078
SBPL/24 h (%)	**R = 0.219 *p* = 0.037**	R = 0.212 *p* = 0.144	R = −0.023 *p* = 0.886
DBPL/24 h (%)	**R = 0.245 *p* = 0.019**	R =0.204 *p* = 0.160	R = 0.162 *p* = 0.305
DIP sys	R = −0.037 *p* = 0.728	R = 0.027 *p* = 0.852	R = −0.113 *p* = 0.476
DIP dia	R = −0.080, *p* = 0.436	R = 0.059 *p* = 0.688	R = −0.241 *p* = 0.124

ARC—active renin concentration, BMI—body mass index, NLR–neutrophil-to-lymphocyte ratio, PLR—platelet-to-lymphocyte ratio, MPV—mean platelet volume, eGFR—estimated glomerular filtration rate according to Schwartz formula, LDL—low-density lipoprotein, HDL—high-density lipoprotein, Alb/Crea ratio—albumin-to-creatinine ratio in urine, SBP—systolic blood pressure, DBP—diastolic blood pressure, MAP—mean arterial pressure, PP–pulse pressure, HR—heart rate, SBPL—systolic blood pressure load, DBPL—diastolic blood pressure load, DIP—blood pressure dipping.

**Table 5:** Diagnostic accuracy of presence of primary hypertension, systolic-diastolic hypertension, and elevated office and ambulatory blood pressure to predict active renin concentration.

Parameter	AUC (95CI)	*p*	Renin Cut-off Value [pg/mL]	Sensitivity (%)	Specificity (%)	ACC (%)
Primary hypertension	0.614 (0.501–0.727)	0.048	17.2	90.2	28.9	0.615
Systolic-diastolic hypertension *	0.704 (0.558–0.850)	0.006	33.9	66.7	73.3	0.636
95c SBP	0.686 (0.575–0.796)	0.001	26.8	68.0	68.2	0.681
95c DBP	0.690 (0.578–0.802)	<0.001	26.8	79.2	58.6	0.638
95c ABPM MAP 24h	0.744 (0.623–0.864)	<0.001	33.9	71.4	72.7	0.725

AUC—area under the curve, ACC—accuracy, 95c SBP—95th percentile of office systolic blood pressure, 95c DBP—95th percentile of office diastolic blood pressure, 95c ABPM MAP 24h—95th percentile of mean arterial pressure during 24 h in ambulatory blood pressure monitoring. *—Patients with primary hypertension.

## Data Availability

The data presented in this study are available upon request from the corresponding author.
